# The Interaction of the Antitumor Complexes Na[*trans*-RuCl_4_ (DMSO)(Im)] and Na[*trans*-RuCl_4_(DMSO)(Ind)] With Apotransferrin: a Spectroscopic Study

**DOI:** 10.1155/MBD.1996.1

**Published:** 1996

**Authors:** Luigi Messori, Felix Kratz, Enzo Alessio

**Affiliations:** 1 Department of Chemistry University of Florence via G. Capponi 7 Florence I-50121 Italy; 2 Institute of Inorganic Chemistry University of Heidelberg Im Neuenheimer Feld 270 Heidelberg D-69120 Germany; 3 Department of Chemistry University of Trieste via L. Giorgieri Trieste I-34127 Italy

## Abstract

The interaction of two antitumor ruthenium(III) complexes,-Na[trans-RuCl_4_(DMSO)(Im)] and Na[trans-RuCl_4_(DMSO)(Ind)]- with human serum apotransferrin (apoTf) was investigated through a number of spectroscopic techniques such as UV-Vis absorption, CD and ^1^H NMR spectroscopy. Interestingly, the hydrolysis profiles of these complexes in a physiological buffer are markedly affected by the presence, in solution, of apoTf suggesting the occurrence of a specific interaction of their respective hydrolysis products with the protein. The formation of stable adducts with apotransferrin has been demonstrated by CD spectroscopy, and additional information obtained through ^1^H NMR of the hyperfine shifted signals. The bound ruthenium(III) species may be detached from these adducts by addition of excess citrate at low pH. The behavior of the investigated ruthenium(III) complexes with apoTf is compared with that of the recently described and strictly related *ru-im* and *ru-ind* antitumour complexes, and discussed in the frame of general strategies of drug targeting.


### THE INTERACTION OF THE ANTITUMOR COMPLEXES Na[*trans*-$RuCl_4$(DMSO)(Im)] AND Na[*trans*-$RuCl_4$(DMSO)(Ind)] WITH APOTRANSFERRIN: A SPECTROSCOPIC STUDY

Luigi Messori*[1], Felix Kratz[2] and Enzo Alessio[3]
[1] Department of Chemistry, University of Florence, via G. Capponi 7, I-50121 Florence, Italy
[2] Institute of Inorganic Chemistry, University of Heidelberg, Im Neuenheimer Feld 270, D-69120 Heidelberg, Germany
[3] Department of Chemistry, University of Trieste, via L. Giorgieri, I-34127 Trieste, Italy

### Abstract.
The interaction of two antitumor ruthenium(III) complexes, -Na[trans-$RuCl_4$(DMSO)(Im)] and Na[trans-$RuCl_4$(DMSO)(Ind)]- with human serum apotransferrin (apoTf) was investigated through a number of spectroscopic techniques such as UV-Vis absorption, CD and $^1H$ NMR spectroscopy. Interestingly, the hydrolysis profiles of these complexes in a physiological buffer are markedly affected by the presence, in solution, of apoTf suggesting the occurrence of a specific interaction of their respective hydrolysis products with the protein. The formation of stable adducts with apotransferrin has been demonstrated by CD spectroscopy, and additional information obtained through $^1H$ NMR of the hyperfine shifted signals. The bound ruthenium(III) species may be detached from these adducts by addition of excess citrate at low pH. The behavior of the investigated ruthenium(III) complexes with apoTf is compared with that of the recently described and strictly related *ru-im* and *ru-ind* antitumour complexes, and discussed in the frame of general strategies of drug targeting.

### Introduction.

After the discovery of the excellent antitumor properties of cisplatin, in the late seventies, large interest has concentrated on the design and the synthesis of other platinum and non-platinum metal complexes to be used for anticancer chemotherapy[1] The main goal of the intense research activity conducted in this field is to prepare new inorganic compounds that are active versus tumor cell lines <u>not sensitive</u> to cisplatin; moreover several attempts are being done to increase the selectivity of these compounds for the target tissue and reduce their systemic toxicity. So, a large number of platinum derivatives as well as several compounds with other metals like ruthenium, titanium, tin, and gold, have been synthesized and tested for anticancer properties[1]. In some cases these compounds demonstrated, in comparison to cisplatin, a different pattern of *in vitro* antitumor activities and a lower systemic toxicity. Unfortunately, only few compounds passed the first phases of the pharmacological evaluation and are now in clinical trials. Complexes that are about entering the first phase of clinical trials are the ruthenium(III) complexes imidazolium *trans*(bisimidazole) tetrachlororuthenate(III) (*ru-im or ICR)* and indazolium *trans*(bisindazole) tetrachlororuthenate(III) (*ru-ind)*, developed by Keppler, for which a good antitumor activity versus several human colon carcinoma cell lines has been reported[1-3]. The corresponding ruthenium(III) compounds developed by the group of Mestroni in which one of the two axial heterocycles -imidazole (Im) or indazole (Ind)- is replaced

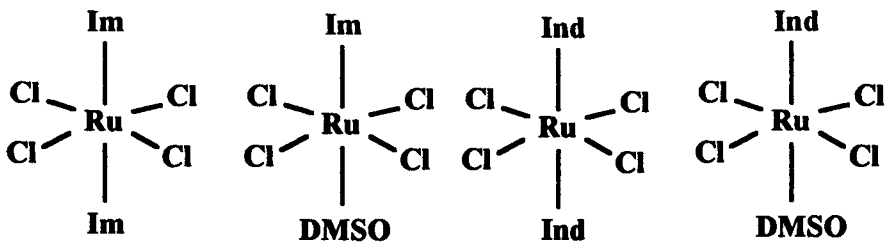

*ru-im* I *ru-ind* II

by a DMSO moiety (respectively Na[trans-$RuCl_4$(DMSO)(Im)] (I) and Na[trans-$RuCl_4$(DMSO)(Ind)]) (II) seem promising[3]. In an effort to improve the antitumor properties of all these ruthenium(III) compounds, we have undertaken an extensive research program aimed at the preparation of conjugates with natural or artificial biomolecules capable to confer selectivity towards the desired biological targets. We started from the consideration that these metal complexes, like several other anticancer compounds, are relatively simple molecules with a limited amount of chemical information and therefore scarce selectivity for the target; this is probably one of the main reasons of their low specificity and of the consequent high toxicity. We thought that this problem could be overcome, at least in part, by adopting appropriate *drug targeting* strategies, for instance by linking these drugs to biological macromolecules endowed with high selectivity for well defined biological targets. Successful targeting strategies are, indeed, those capable of attaining a high local concentration of the drug at the level of a certain organ or tissue while keeping its concentration low in the other districts[4-6].

Our drug targeting project stems on the utilization of transferrin as the carrier biomolecule. The choice of transferrin is based on the following reasons: i) transferrin is physiologically present in the biological fluids and therefore virtually devoid of immunogenicity[7]; ii) transferrin exhibits good binding properties for metal ions and metal complexes[8]; iii) the protein enters cells easily through the transferrin cycle comprising protein recognition by a specific cell surface receptor and internalization into an endosomic compartment[9]; iv) the expression rate of the transferrin receptors is markedly enhanced in fast proliferating cells so that the uptake rate of the drug-protein adducts by the tumor cells is high[10,11]; v) the fact that the protein, during its intracellular cycle, passes through an acidic compartment gives us the chance of preparing acid-labile adducts capable of releasing the drug inside the cell where the pH is low[12].

Strategies of drug targeting using transferrin as carrier were first reported during the eighties by Faulk et al. who prepared and tested transferrin conjugates of doxorubicin.[13-15] We have resumed early Faulk's ideas and attempted to apply them to these ruthenium(III) complexes. In a previous study we already investigated the interaction of *ru-im* and *ru-ind* with apotransferrin[16]. We report here the analysis of the interaction of complexes I and II with apotransferrin. Interestingly, the latter complexes are far more soluble in water and therefore easier to be handled with.

### Materials and Methods

Complexes I and II were synthesized as previously described[17]. Human serum apotransferrin was purchased from Sigma Chemical Company and further purified according to standard procedures[18]. In all the experiments a physiological buffer was used so that the final salt concentrations were always 0.004 M $NaH_2PO_4$, 0.1 M NaCl and 0.025 M $NaHCO_3$ with pH 7.4.

The absorption spectra were recorded both on a Cary 17D and a Cary 3 spectrophotometer; the CD spectra on a JASCO 200C spectropolarimeter. The $^1H$ NMR

experiments were performed on a Bruker MSL 200 spectrometer operating at 4.7 T, typically using 0.5 ml. samples in 5 mm tubes. In order to improve the detection of the broad hyperfine shifted signals short relaxation delays of the order of 50-100 ms were used. Each spectrum typically consisted of 32,000-64,000 scans. The FIDs of 8 K data points each, were processed using line broadening factors of the order of 10-30 Hz.

### Results

***Hydrolysis of I and II within the physiological buffer: the electronic spectra.***

Complexes **I** and **II** are relatively stable under acidic conditions but rapidly hydrolyze at neutral or alkaline pH. Hydrolysis can be easily followed by monitoring the absorption spectra in dependence of time as already reported for complex **I** [17]. Under physiological conditions hydrolysis appears to be at least a two-step process, the first step corresponding to the loss of a first chloride group and the second step to further hydrolytic events. The spectra of **I** and **II**, recorded at increasing time intervals after mixing with the buffer, at 25°C, are shown in Figure 1A and 2A, respectively. The initial spectra are characterized by an intense band at 385 nm plus a shoulder at 445 nm in the case of **I** and by a band at 395 nm plus a shoulder at 465 nm in the

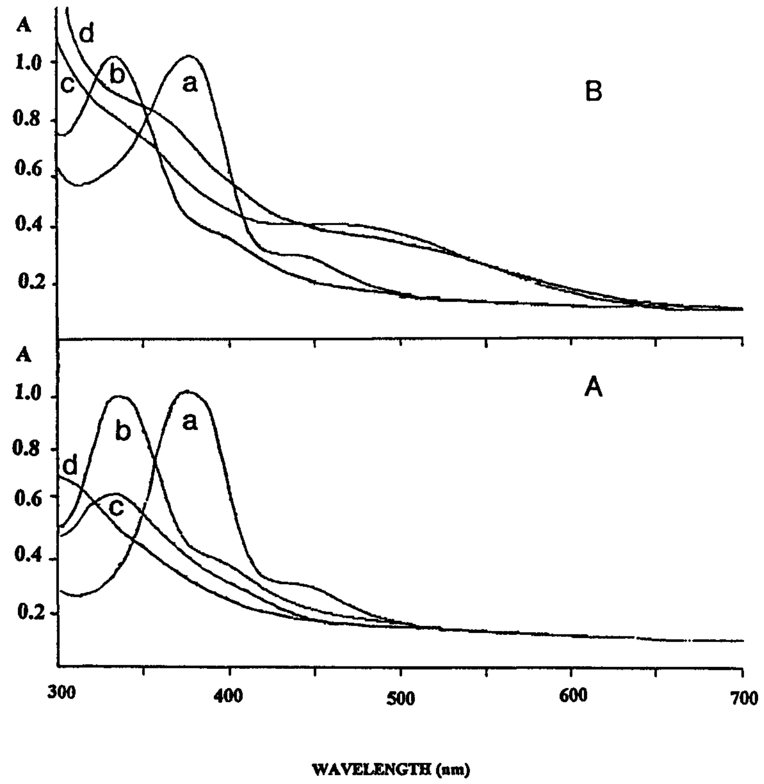

Figure 1 Hydrolysis of **I** in the physiological buffer in the absence (A) and in the presence (B) of apoTf as followed through absorption spectroscopy. Traces **a**, **b**, **c** in Figure **A** were respectively recorded 5, 30 and 180 minutes after mixing. Trace **d** is the spectrum recorded the day after. Traces **a**, **b**, **c** in Figure **B** were recorded at 3, 10 and 30 minutes after mixing. Trace **d** was recorded the day after. Conditions: [**I**] was $2x10^{-4}$ M; protein concentration $1x10^{-4}$ M. A physiological buffer containing 0.004 M $NaH_2PO_4$, 0.1 M NaCl and 0.025 M $NaHCO_3$ was used, with pH 7.4. T= 25°C.

case of **II**. Within few minutes the two main bands and the respective shoulders quickly decrease in intensity and eventually vanish whereas a new intense band appears in both cases around 345 nm. The intensity of the new band reaches a maximum after about half an hour, then, on turn, starts decreasing and vanishes. The final spectrum of **I** in the buffer, recorded several hours later, is characterized by a broad band in the UV with a shoulder around 320 nm. In the case of **II**, a marked increase in the intensity of the whole spectrum is observed with time; a new, broad band also develops around 570 nm.

*Hydrolysis of I and II in the presence of ApoTf- the electronic spectra.*

In order to ascertain whether complexes **I** and **II** interact with apotransferrin we decided to repeat the above hydrolysis experiments within the buffer in the presence of apoTf. The family of spectra obtained upon hydrolysis of **I** and **II** in the presence of the protein are respectively shown in Figure 1B and 2B.

The first part of the hydrolysis of both complexes within the buffer as monitored through absorption spectroscopy is not significantly affected by apoTf even if the hydrolysis rates are significantly enhanced. During the second phase of the hydrolysis, however, the absorption spectra, differ significantly from those obtained in the absence of apoTf . The final spectra are invariably characterized by a broad, relatively intense transition in the visible, located around 500

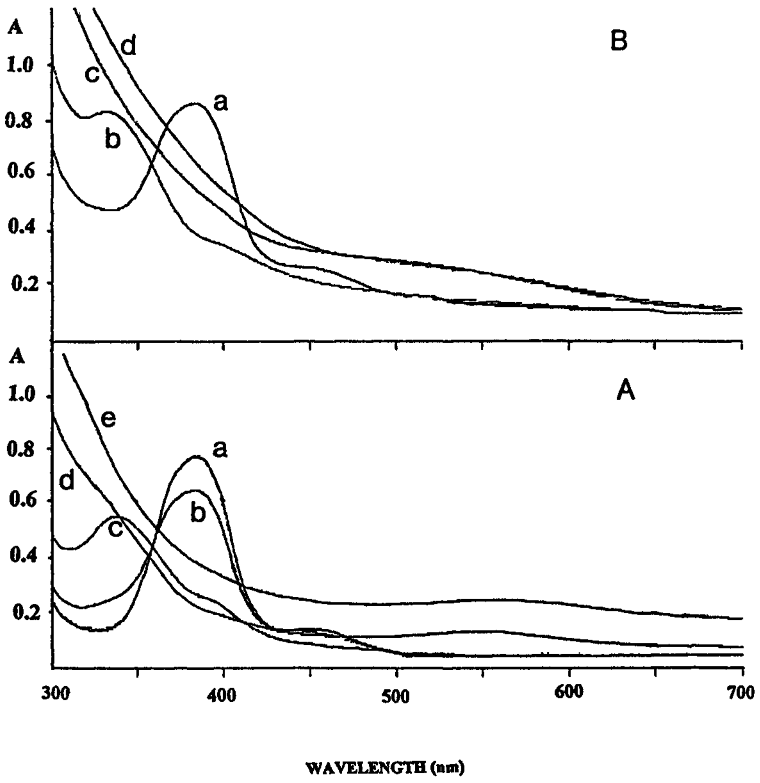

Figure 2 Hydrolysis of **II** in the buffer in the absence (A) and in the presence (B) of apoTf as followed through absorption spectroscopy. Traces **a**, **b**, **c** and **d** in Figure A were respectively recorded 3, 30 and 120 and 240 minutes after mixing. Trace **e** is the spectrum recorded the day after. Traces **a**, **b**, **c** in Figure B were recorded at 3, 10 and 30 minutes after mixing. Trace **d** was recorded the day after.Conditions: [**II**] was $2x10^{-4}$ M; protein concentration $1x10^{-4}$ M. A physiological buffer containing 0.004 M $NaH_2PO_4$, 0.1 M NaCl and 0.025 M $NaHCO_3$ was used with pH 7.4. T= 25°C.

nm in the case of complex I and around 540 nm in the case of complex II. These results indicate that both complexes or, more precisely, their hydrolysis products *do* interact with apotransferrin and that the interaction occurs in concomitance with the second hydrolytic process.

*Interaction of I and II with apoTf- the CD spectra.*

To better elucidate the formation of specific adducts between complexes I and II and apoTf we analyzed the final hydrolysis products obtained both in the absence and in the presence of apoTf, by CD spectroscopy. CD spectroscopy is a valuable tool to demonstrate the specific binding of small metal complexes, with transitions in the visible region, to chiral biomolecules[19]. Whereas, as expected, no CD band was observed in the case of the complexes dissolved in the buffer, the spectra of the samples dissolved in buffered apoTf solutions, at a 2:1 ratio, showed characteristic CD transitions in the visible region (Figure 3). More in detail, the spectrum of I plus apoTf consists of two positive transitions at 370 and 480 nm; that of II plus apoTf shows two positive transitions at 375 and 495 nm. The detection of these CD transitions provides unambiguous evidence for a tight interaction between the ruthenium(III) chromophore and the chiral macromolecule in both cases. Interestingly the CD spectra develop a few hours after mixing in concomitance with the second hydrolytic process of both complexes.

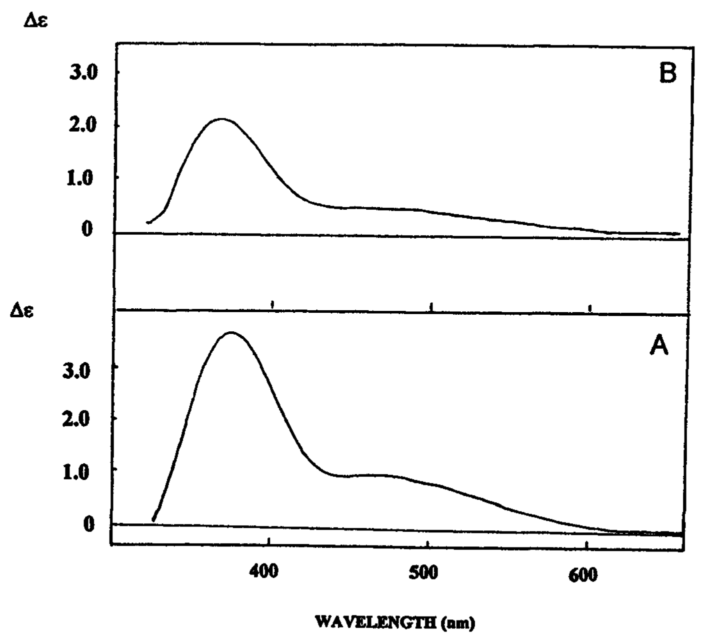

Figure 3 CD spectra in the visible region of I plus-apoTf (A) and II plus-apoTf (B). At 25 °C both spectra fully develop about 60-90 minutes after mixing. The samples of Figures 1B and 2B were used.

The CD spectra differ markedly from those, previously reported, of ruthenium(III) transferrin[20] ruling out that the bound species is the bare ruthenium(III) ion. Moreover, the fact that the CD spectra of I plus apoTf and II-plus apoTf, although similar, are different in shape and intensity suggests that in the protein-bound species the heterocycle remains attached to the ruthenium(III) ion.

Remarkably, the characteristic CD transitions are abolished by treating these adducts with excess citrate (10 mM) at low pH (pH ≈ 4) indicating that the interaction of both ruthenium(III) complexes with apoTf is reversible and acid labile.

*Interaction with apoTf- $^1H$ NMR spectra*

Ruthenium(III) is paramagnetic and is a reasonably appropriate probe for $^1$H NMR spectroscopy of paramagnetic species[21]. The paramagnetic $^1$H NMR spectra of **I** and **II** are characterized by a few, relatively narrow hyperfine signals spread within the upfield region. The spectra are shown in Figure 4A and 4B. The assignment of the $^1$H NMR spectrum of **I** has been previously reported[17]. The spectrum of **II** is poorer and yet not assigned (Figure 4A and B); it is straightforward, however, to attribute the intense (and virtually only) feature observed at -14 ppm to the methyl groups of the ruthenium(III)-bound DMSO moiety.

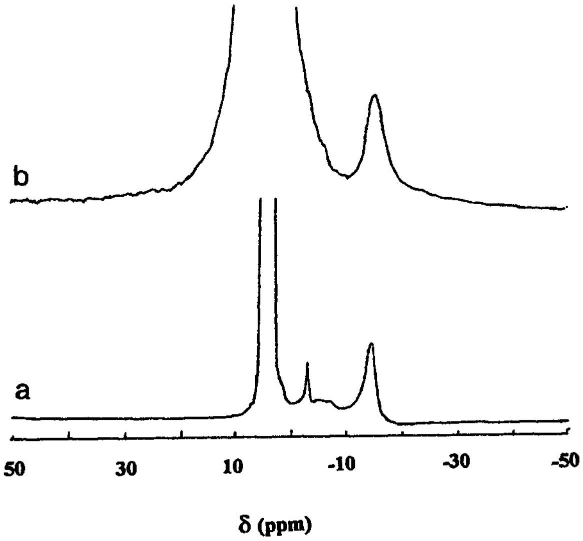

Figure 4 $^1$H NMR spectra of **I** (a) and **II** (b) freshly dissolved in $D_2O$.

Conditions: 10 mM **I** or **II**;T=25°C.

Given the presence of these characteristic hyperfine signals in the respective spectra, we thought that $^1$H NMR spectroscopy might represent a valuable technique to better characterize the interaction of our ruthenium(III) complexes with diamagnetic apoTf.

*a) I plus apoTf*

The 200 MHz $^1$H NMR spectra of apoTf at increasing time intervals after addition of 2 equivalents of **I**, at 25 °C, are shown in Figure 5. The spectra, all recorded within 4 hours after mixing, clearly show that the signals of the free complex, and in particular the intense signal of DMSO at -14 ppm, quickly disappear with time. Simultaneously, new hyperfine signals appear both upfield and downfield, -signals labeled with an asterisk in figure 5-. This behavior suggests that the original complex breaks down rapidly and its hydrolysis products bind the protein. The new signals should correspond to those of the adduct of apoTf with the hydrolysis product of **I**. The final spectrum is stable for hours, and is markedly different from that obtained upon reaction of apoTf with two equivalents of **II** (see below).

*b) II plus apoTf*

Similar $^1$H NMR studies were performed to monitor the interaction of **II** with apoTf. The

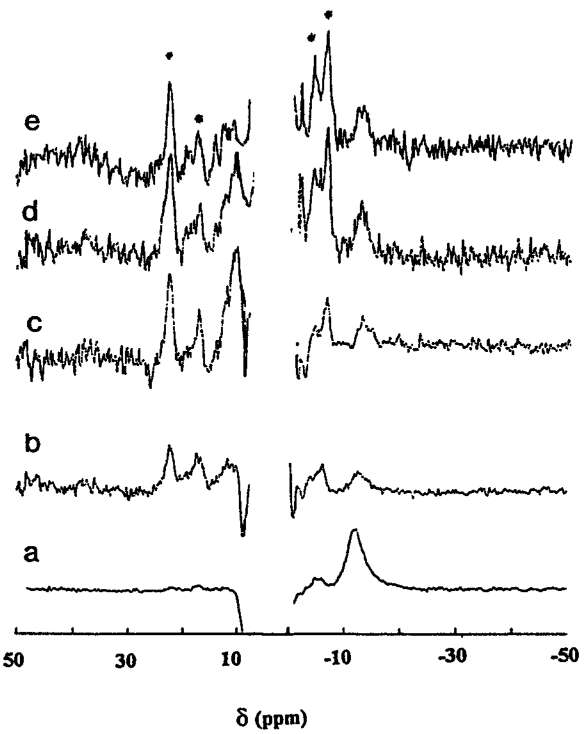

Figure 5 $^{1}H$ NMR spectra of apoTf plus two equivalents of I at different times after mixing in the deuterated physiological buffer. The spectra were respectively recorded at 15' (a), 60' (b), 120' (c), 180' (d), and 240' (e) after mixing. Conditions 2 mM [I] in the deuterated buffer with 1mM apoTf. T=25°C.

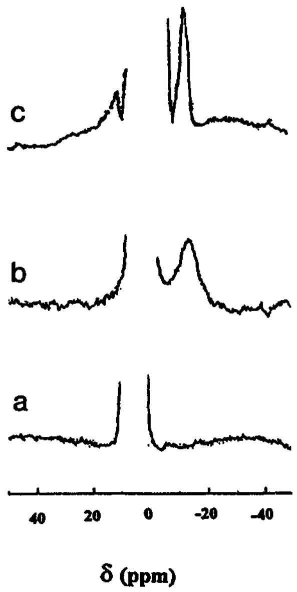

Figure 6 $^{1}H$ NMR spectra of 1 mM apoTf in the deuterated physiological buffer alone (a), and 30' (b) and 12 hours (c) after addition of two equivalents of II in the physiological buffer.

spectra are shown in Figure 6. Upon addition of two equivalents of II to a solution containing 1mM apoTf an intense, broad hyperfine signal at -15 ppm is observed; within few hours the spectrum transforms into another spectrum characterized by an intense sharper resonance at -14 ppm. This spectrum is markedly different from that of I plus-apoTf, implying a somewhat unequivalent structure for the two adducts.

### Discussion.

*Targeting of antitumor complexes.*

The concept of *drug targeting* may be of great help when dealing with drugs characterized by a narrow therapeutic index. The main goal of drug targeting strategies is to obtain a more favorable biodistribution of a drug within the organism, thus reducing its systemic toxicity. Even if the selectivity of transferrin conjugates - owing to the large distribution of the transferrin receptors in the organism[9]- may not be as high as that of monoclonal antibodies conjugates, transferrin conjugates show a number of properties that make them suitable for drug targeting experiments. Indeed, by using transferrin as carrier, it is possible, in principle, to achieve a preferential uptake of the drug by those tissues that exhibit a large demand of iron, in other words by fast replicating tissues like cancer tissues. Previous reports on the use of transferrin as a carrier of cytostatic drugs or toxins are in the literature[12-15]. We have attempted to apply this strategy to a series of ruthenium(III) complexes with a structure resemblant that of cisplatin.

*Binding of **I** and **II** to apoTf.*

The preliminary -and indispensable- step of this kind of studies is represented by the preparation and characterization of stable adducts between the metal complexes and transferrin and by the assessment of their acid lability[16]. We have reported here on the interaction of **I** and **II** with apotransferrin under physiological conditions, as monitored by spectroscopic methods. The absorption spectroscopy studies allowed us to describe in detail the hydrolysis processes that occur when the two ruthenium(III) complexes are exposed to a neutral environment. In the absence of transferrin, both complexes roughly follow the same hydrolysis pathway comprising a "fast phase" that reaches completion within about half an hour and a "slow phase" that takes hours. When repeating the same reaction in the presence of apoTf significant variations are observed during the "slow phase", ascribed to a direct interaction of the hydrolysis products of both complexes with the protein. Following these preliminary indications, we have demonstrated the occurrence of a specific interaction of both ruthenium(III) compounds with apoTf by means of CD spectroscopy. The CD spectra of the drug/protein adducts, obtained at a 2:1 stoichiometry, are similar but not identical suggesting that the heterocycle remains attached to the ruthenium(III) ion. Moreover, we have demonstrated that the obtained drug/protein adducts, like the analogous adducts with *ru-im* and *ru-ind*[16], may be broken down by acification and complexation by citrate; this finding is of particular interest since *in vivo* transferrin enters a cytoplasmic low pH compartment where the loaded drug may be released.

Further, independent information on the protein-binding process has been gained by $^1$H NMR spectroscopy, monitoring the hyperfine signals of the ruthenium(III) ligands. In the case of **I** it is apparent that the signals of the free complex rapidly decrease in intensity after mixing and that a new set of hyperfine signals appear, both upfield and downfield. Such behavior clearly reflects the hydrolysis of the free complex in the physiological buffer and the subsequent binding of the hydrolyzed species to the apoprotein. The $^1$H NMR behavior of **II** is apparently different: upon hydrolysis the intense signal, attributed to the methyl groups of ruthenium(III)-bound DMSO changes slightly in shape and position, suggesting that DMSO remains attached to ruthenium(III) in the protein bound species.

*Comparison with ru-im and ru-ind.*

Upon comparing the reactivity patterns of **I** and **II** with those of *ru-im* and *ru-ind*, one can state that all these compounds roughly behave in a similar way in that all bind apotransferrin tightly, probably around the metal binding region, with a 2:1 drug/protein stoichiometry. The possibility of forming stable adducts with apotransferrin is promising in view of targeting strategies to tumor tissue; remarkably, all these adducts can release the bound ruthenium(III) species upon exposure to citrate in a slightly acidic environment, a circumstance which is met also in vivo. There are however some important, distinctive features between the two classes of compounds, such as the different kinetics of hydrolysis and protein binding and the different water solubility. The latter feature, in particular, renders the DMSO containing compounds attractive for future clinical applications.

*Concluding Remarks*

In the frame of a larger project on specific targeting of antitumor metal complexes exploiting transferrin as a carrier we have investigated the interaction of two water-soluble antitumor ruthenium(III) complexes, **I** and **II**, with the apoprotein. Noticeably the protein binds at least two equivalents of both complexes. The binding process has been characterized through a host of spectroscopic techniques comprising absorption, CD and $^1H$ NMR spectroscopies. Protein binding takes place only when the complexes have undergone the first step of their hydrolytic process. The resulting adducts are stable for several hours under physiological conditions; addition of excess citrate at low pH causes detachment of the bound ruthenium(III) species. Apparently these adducts display sufficient requirements as to be tested *in vitro* and *in vivo* for antitumor activity.

**References**

1. *Metal Complexes in Cancer Chemoterapy*, B.K. Keppler, (ed.) Weinheim: VCH, 1993.
2. B.K. Keppler, M. Henn, U.M. Juhl, M.R. Berger, R. Niebl, and F.F. Wagner, *Prog. Clin. Biochem. Med.* **10**, 41 (1989).
3. G. Mestroni, E. Alessio, G. Sava, S. Pacor, M. Coluccia and A. Boccarelli, *Metal Based Drugs* **1**, 41 (1994).
4. J.E. Roulston, *Chem. Brit.* 770 (1993).
5. I.S. Trowbridge and D.L. Domingo, *Nature* **294**, 171 (1981).
6. P.A. Trail, D. Willner, S.J. Lasch, A.J. Henderson, S. Hofstead, A.M. Casazza, R .A. Firestone, I. Hellstrom and K.E. Hellstrom *Science*, 212 (1993).
7. W.P. Faulk, H. Harats, J.A. McIntyre, A. Berczi, I.L. Sun. and F.L. Crane, *Am. J. Reprod. Immunol.* **21**, 151 (1989).
8. D.C. Harris and P. Aisen, in *Iron Carriers and Iron Proteins* (T.M. Loehr, ed.) pp.239-351, VCH Publishers Inc. New York (1989); *ibidem* P. Aisen, pp. 353- 371; A. Butler and H. Eckert, *J. Am. Chem. Soc.* **111**, 2802 (1989).
9. R.R. Crichton and R.J. Ward, *Biochemistry* **31**, 11255 (1992).
10. U. Testa, E. Pelosi and C. Peschle, *Crit. Rev. Oncogen.* **4**, 241 (1993).
11. M. Cazzola, G. Bergamaschi, L. Dezza . and P. Arosio, *Blood*, **75**, 1903 (1990).
12. H.H. Wellhoner, D.M. Meville, K. Srinvasacher and G. Erdmann *J. Biol. Chem.* **266**, 4309 (1991).
13. W.P. Faulk, H. Harats and A. Berczi, in *Oxidoreduction at the Plasma Membrane* (F.L. Crane, J.D. Morre and H. Low, eds.) vol. 1, pp.205-224, CRC Press, Inc. Boca Raton, FL (1990).
14. K. Barabas, J. A. Sizensky and W.P. Faulk, *J. Biol. Chem.* **267**, 9437 (1992).
15. W.P. Faulk, B.L. Hsi and P.J. Stevens, *Lancet* **2**, 390 (1980).
16. F. Kratz, M. Hartmann, B.K. Keppler. and L. Messori, *J. Biol. Chem.* **269**, 2581 (1994).
17. E. Alessio, G. Balducci, A. Lutman, G. Mestroni, M. Calligaris and W.M. Attia, *Inorg. Chim. Acta* **203**, 205 (1993).
18 G.W. Bates and M.R. Schlabach, *J. Biol. Chem.* **250**, 2177 (1975).
19. S.F. Mason, *Molecular Optical Activity and the Chiral Discrimination*, Cambridge University Press, Cambridge, UK (1982).
20. F. Kratz and L. Messori *J. Inorg. Biochem.* **49**, 79 (1993).
21. I. Bertini and C. Luchinat, *NMR of Paramagnetic Molecules in Biological Systems* Benjamin Cummings, Menlo Park, California (1986).

**Received: August 21, 1995 - Accepted: October 9, 1995 - Received in revised camera-ready format: November 29, 1995**

